# Un énorme adénome de la prostate

**DOI:** 10.11604/pamj.2018.30.256.12142

**Published:** 2018-08-06

**Authors:** Abdelilah El Alaoui, Hicham El Boté

**Affiliations:** 1 Service d’Urologie A, Hôpital Ibn Sina, CHU Rabat, Maroc

**Keywords:** Adénome, prostate, Maroc, Adenoma, prostate, Morocco

## Abstract

We here report the case of a 75 year old patient, with lower urinary tract obstructive syndrome such as dysuria, nocturnal pollakiuria and dribbling. Clinical examination showed good general condition by examining the rectum with the finger, soft, painless prostate increased in volume with obliteration of the median sulcus. PSA level was 2,1ng/ml, prostatic weight was 240 g on ultrasound. The patient underwent surgery: transvesical adenomectomy with extraction of a giant adenoma weighing 246 g. Histologic examination showed adenomyofibroma.

## Image en médecine

Il s'agit d'un patient âgé de 75 ans, qui présente un syndrome obstructif du bas appareil urinaire fait de dysurie, pollakiurie nocturne et miction goutte à goutte. L'examen clinique trouve un patient en bon état général; au toucher rectal, la prostate est souple, indolore et augmenté de volume avec effacement du sillon médian. Le PSA est à 2,1ng/ml, le poids de la prostate a l'échographie est estimé à 240g. Le patient est opéré: adénomectomie par taille vésicale avec extraction d'un énorme adénome qui pèse 246g. L'histologie est en faveur d'un adénomyofibrome.

**Figure 1:**
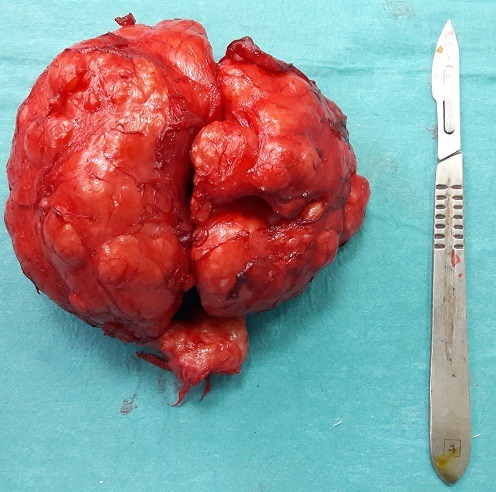
Un énorme adénome de la prostate

